# P-2201. The Burden of Influenza on Health-Related Quality of Life and Work Productivity During the First Week of Infection Among US Adults: An Interim Analysis of a Nationwide Prospective Study

**DOI:** 10.1093/ofid/ofaf695.2364

**Published:** 2026-01-11

**Authors:** Tianyan Hu, Alon Yehoshua, Joseph C Cappelleri, Meghan Gavaghan, Manuela Di Fusco, Xiaowu Sun

**Affiliations:** Pfizer Inc., New York, New York; Pfizer Inc., New York, New York; Pfizer Inc., New York, New York; Pfizer Inc., New York, New York; Pfizer Inc, New York, New York; CVS Health, Woonsocket, Rhode Island

## Abstract

**Background:**

This study characterized Health-Related Quality of Life (HRQoL) and Work Productivity and Impairment (WPAI) among outpatient symptomatic adults during the first week of a test-confirmed influenza infection in the US in the 2024/25 respiratory season.

**Methods:**

Symptomatic adults with test-confirmed influenza infection were enrolled at CVS Health between 10/24/2024-4/15/2025 (CT.gov: NCT05160636). Questionnaires on socio-demographics and clinical characteristics were administered to participants via an online survey platform at enrollment. Validated instruments (EQ-5D-5L, WPAI-GH) were used to assess HRQoL and WPAI for the pre-infection baseline (through recall at enrollment) and at Week 1. Outcomes were summarized using descriptive statistics for each time point and compared between time points using paired t-tests.

**Results:**

Of 720 participants, mean age was 42.0 (SD: 13.0), 73.8% were female, 47.1% had at least one comorbidity, and 588 (81.7%) were employed at baseline. The mean (SD) of EQ-Visual Analogue Scale (EQ-VAS) and Utility Index (UI) scores were 89.7 (SD: 7.8) and 0.96 (SD: 0.07), respectively, at the pre-infection baseline. Both scores statistically significantly decreased to 84.9 (SD: 11.0) for EQ-VAS and 0.94 (SD: 0.10) for UI scores at Week 1, with a mean change of -4.9 (p< 0.001) for EQ-VAS, and -0.02 (p< 0.001) for UI scores. Relative to baseline, the mean changes at Week 1 were all statistically significant, with 55.0% (p< 0.001) in work productivity time loss, 33.2% (p< 0.001) in absenteeism, 42.2% (p< 0.001) in presenteeism, and 45.3% (p< 0.001) in activity impairment.

**Conclusion:**

Influenza infections negatively impacted the health-related quality of life and especially work productivity among US adults during the 2024/25 season up to the first week of infection. These findings underscore the broad and lasting consequences of influenza infections and help raise awareness of the value of preventive measures such as influenza vaccination.

**Disclosures:**

Tianyan Hu, PhD, Pfizer: Salary|Pfizer: Stocks/Bonds (Public Company) Alon Yehoshua, PharmD, MS, Pfizer Inc: Employee of Pfizer and may hold stock or stock options of Pfizer Joseph C. Cappelleri, PhD, Pfizer Inc.: Employee|Pfizer Inc.: Stocks/Bonds (Public Company) Meghan Gavaghan, MPH, Pfizer Inc.: Employee Manuela Di Fusco, PhD, Pfizer Inc: Employee of Pfizer and may hold stock or stock options of Pfizer Xiaowu Sun, PhD, CVS Health: Employee|CVS Health: Stocks/Bonds (Public Company)

